# Knowledge and Perceptions of Pediatric Hearing Loss among University Students in Jordan

**DOI:** 10.1055/s-0046-1818564

**Published:** 2026-04-30

**Authors:** Reeman Marzouqah, Rama Alasir, Laila Qanawati, Yazan Gammoh

**Affiliations:** 1 Department of Communication Sciences and Disorders, College of Communications, California State University, Fullerton (CSUF), Fullerton, CA, United States; 2 Department of Audiology and Speech Pathology, Faculty of Allied Medical Sciences, Al-Ahliyya Amman University, Amman, Amman Governorate, Jordan; 3 Department of Optometry Science, Faculty of Allied Medical Sciences, Al-Ahliyya Amman University, Amman, Amman Governorate, Jordan

**Keywords:** pediatric hearing loss, university students, Jordan, awareness, audiology education, early detection

## Abstract

**Introduction:**

Early identification of pediatric hearing loss is vital to reduce long-term developmental consequences; however, awareness among future healthcare professionals remains limited in many low- and middle-income countries.

**Objective:**

To assess the knowledge and perceptions of university students in Jordan regarding pediatric hearing loss.

**Methods:**

We conducted a cross-sectional study using a questionnaire that evaluated the students' awareness of the common causes and risk factors for hearing loss in children, perceptions of early intervention, and self-reported confidence in identifying and referring potential cases. The awareness and perception items were grouped into two categories, and misconception rates were calculated as the proportion of respondents answering *agree* or *strongly agree* to statements that were factually incorrect. Chi-square tests were conducted to examine associations between participant demographics and response patterns, with statistical significance set at *p* < 0.05.

**Results:**

A total of 411 students participated (response rate: 84.4%); most were aged from 18 to 25 years (81%) and female (53%), with 65% of them enrolled in the Faculty of Allied Medical Sciences, Al-Ahliyya Amman University, Jordan. Misconceptions were more common in awareness items (mean: 30.4%), with the highest rate observed for the belief that neonatal meningitis or seizures do not cause hearing loss (44%). Perception items showed lower misconception rates (mean :13.4%), with the highest being that parental suspicion should not influence clinical opinion (26%). The Chi-squared analysis revealed that higher awareness was significantly associated with year of study ( *p* < 0.001), major ( *p* < 0.001), and family history ( *p* < 0.05).

**Conclusion:**

Despite their positive attitudes, students showed limited clinical knowledge. Strengthening audiology education in health science university programs may improve early detection and intervention outcomes in Jordan.

## Introduction

Hearing loss is the fourth leading cause of disability globally, and its prevalence continues to rise. 1 An estimated 430 million people with moderate-to-severe hearing loss need rehabilitation services. 2 Often described as an *invisible disability* , hearing loss lacks overt physical signs, leading to its underestimated impact on individuals and society. 3 Its consequences extend beyond auditory function, negatively affecting cognitive performance, communication ability, and quality of life. 4 Despite these widespread effects, hearing loss remains underprioritized in global health funding and policy. 5

A disproportionate burden of hearing loss is carried by low- and middle-income countries, including Jordan, where access to ear and hearing care remains limited. 6 This gap in services contributes to stark disparities in intervention, as only 17% of individuals who could benefit from a hearing aid use one. 7 In Jordan, the combination of genetic, environmental, and healthcare-related factors contributes to the rising prevalence of hearing loss. 8 9 Congenital hearing loss in Jordan, for example, affects approximately 15 per 1 thousand live births, which is substantially higher than the 1 to 4 cases per 1 thousand live births reported in many high-income countries. 8 This elevated rate has been linked to the high prevalence of consanguineous marriages, which increase the likelihood of inherited hearing impairment. 9 Compounding these genetic risks are systemic challenges, including limited access to newborn hearing screening and a shortage of trained professionals in hearing healthcare. 10 Although national initiatives have aimed to expand early detection and intervention services, significant gaps persist in public awareness and in the integration of hearing care into primary healthcare systems. 11 Similar challenges have been noted in diaspora contexts, in which Arabic-speaking populations in North America face linguistic and cultural barriers to equitable hearing healthcare. 12

In light of these ongoing barriers, it is important to examine the knowledge and attitudes of students in health science disciplines such as medicine and pharmacy. These future professionals play a key role in early identification and referral, which are critical to prevent long-term communication and developmental issues. 13 Studies conducted in countries such as Nigeria, 14 Samoa, 13 India, 15 and Saudi Arabia, 16 have reported limited awareness among health sciences students about pediatric hearing loss, particularly regarding risk factors. These studies 13 14 15 16 have also found that students often lacked confidence in referring or managing cases effectively, suggesting a shared educational gap across these contexts. Despite Jordan's high burden of pediatric hearing loss, research in this field is limited.

Thus, the present study assessed the knowledge and attitudes of pharmacy and medical sciences students at Al-Ahliyya Amman University, in Jordan, regarding pediatric hearing loss. The findings may inform curriculum development and contribute to improve hearing health education in similar university programs.

## Methods

### Study Procedure

We conducted a cross-sectional study in which pharmacy and medical sciences students at Al-Ahliyya Amman University completed the “Awareness and Perceptions of University Students” questionnaire. The tenets of the Declaration of Helsinki were followed, and ethical approval to conduct the study was obtained from the Scientific Research Ethics Committee at Al-Ahliyya Amman University (IRB: AAU/4/4/2023-2024). The participants provided written informed consent prior to inclusion, and they had the right to withdraw from the study at any time.

The research assistants approached the participants as they entered the Faculty of Pharmacy and Faculty of Allied Medical Sciences faculty building at Al-Ahliyya Amman University. To minimize sampling bias, a systematic sampling method was employed, in which every third student entering the building was invited to participate. Participants completed the questionnaire with the assistance of research assistants, which took 10 to 15 minutes. Upon completion, all questionnaires were de-identified, collected by the research team, and securely stored in the faculty's office locker for data entry and analysis.

The questionnaire was originally developed in previous research 14 and has subsequently been applied in studies on pediatric hearing loss. 20 For the present study, six faculty experts in audiology, speech-language pathology, and public health reviewed the adapted questionnaire to establish face and content validity. After the consultation with experts, the questionnaire was slightly changed so that the reference to the nationality of children in question 1 was removed, as children in Jordan represent diverse national backgrounds, and question 2 was expanded into separate items to enhance clarity while retaining the original wording. The revised questionnaire was then piloted with 10 university students who were not included in the final sample. These students reported that the items were clear, relevant, and easy to understand, and they also provided feedback on logistical aspects, including recommendations for the best campus location to reach as many students as possible. Since English is the language of instruction at the university, no translation was required, and pilot testing confirmed that students did not encounter difficulties with comprehension.

The participants were first asked about their demographic information, including gender, age, year of study, major, and history of hearing loss. The questionnaire consisted of 10 items rated on a 5-point Likert scale, with responses ranging from *strongly agree* to *strongly disagree* . The participants marked an “X” to indicate their chosen response. The questionnaire assessed the participants' awareness of the causes and interventions for pediatric hearing loss, as well as their perceptions toward its seriousness and management. The items were grouped into two domains: *awareness* , which included statements addressing biomedical causes, risk factors, and clinical management, and *perception* , which included statements reflecting attitudes and beliefs about the importance, urgency, and detectability of hearing loss. For the analysis, a misconception rate was calculated for each item by adding the proportion of participants who answered *agree* or *strongly agree* to statements that were factually incorrect. This method enabled a comparison of misconception prevalence between the awareness and perception domains.

### Statistical Analyses

Data was entered using Microsoft Excel (Microsoft Corp.) spreadsheets, and data analysis was performed using the IBM SPSS Statistics for Windows (IBM Corp.) software, version 25.0. Descriptive statistics were expressed as numbers and percentages. The Chi-squared test was used to identify demographic factors that were linked to correct responses to the statements in the questionnaire. A sample size of 361 students was deemed representative of the pharmacy and medical students' population at the university, assuming a 95%CI, a margin of error of ± 5%, and a replacement rate of 10%. 17 Statistical significance was set at *p* < 0.05.

## Results

### Sample Characteristics

A total of 411 students participated in the study out of the 487 students who were approached during the data collection stage, yielding a response rate of 84.4%. Table 1 depicts the demographics of the study sample. Most students (53%) were women, aged between 18 and 25 years (81%), and reported no history of hearing loss (78.3%). Most (65%) were students of the Faculty of Allied Medical Sciences, and many of these (31.1%) were in the fifth year of their studies.

**Table 1:** Demographic profile of the study sample (N = 411)

Variable	Category	n (%)
**Gender**	Male	193 (47%)
	Female	218 (53%)
**Age group (years)**	18–25	333 (81%)
	> 25	78 (19%)
**Year of study**	Year 1	74 (18%)
	Year 2	40 (9.7%)
	Year 3	54 (13.1%)
	Year 4	115 (28.1%)
	Year 5	128 (31.1%)
**Major**	Pharmacy	144 (35%)
	Medical Sciences	267 (65%)
**Diagnosis of hearing loss and/or family history of hearing loss**	Yes	89 (21.7%)
	No	322 (78.3%)

### Questionnaire Results

The responses to the questionnaire are summarized in Table 2 . Most participants demonstrated strong awareness of the seriousness of pediatric hearing loss, with 90% disagreeing that it is not a serious problem. Most students also supported early detection and intervention, with 77% disagreeing that babies should wait until they are older for hearing testing. However, uncertainty was evident in several areas, particularly regarding neonatal jaundice (46%: neutral), ototoxic medications such as gentamycin (44%: neutral), and maternal noise exposure during pregnancy (37%: neutral). Deficits in knowledge were observed for key risk factors, and many students undervalued parental concern in clinical decision-making.

**Table 2:** Responses to questionnaire statements (N = 411)

Statement	Strongly disagree (%)	Disagree (%)	Neutral (%)	Agree (%)	Strongly agree (%)
1. Hearing loss is not a serious problem in children	58	32	5	4	1
2. Craniofacial anomalies (e.g., Down's syndrome) do not cause hearing loss	11	34	28	22	5
3. Intrauterine infections (e.g., cytomegalovirus and rubella) do not cause hearing loss	14	24	30	22	10
4. Low birth weight (< 2.5 kg) does not cause hearing loss	12	22	34	29	3
5. Neonatal jaundice does not cause hearing loss	9	19	46	24	2
6. Neonatal meningitis/seizures does not cause hearing loss	16	22	18	30	14
7. Birth asphyxia does not cause hearing loss	13	25	24	27	11
8. Aminoglycosides such as gentamycin will not harm a baby's hearing	13	23	44	15	5
9. A mother's exposure to loud noise in pregnancy will not affect a baby's hearing	11	19	37	22	11
10. Babies with hearing loss cannot be accurately detected at birth	24	24	20	26	6
11. Babies with hearing loss cannot be helped until they are older	29	39	18	11	3
12. Hearing aids are unsuitable for babies	14	32	34	16	4
13. Parental suspicion of hearing loss should not influence a clinical opinion	26	27	21	21	5
14. Babies can wait until they are older before testing for hearing loss	45	32	13	10	0
15. Hearing loss is not that important because it does not kill	56	24	8	10	2

As shown in Table 3 , misconceptions were more prevalent in items assessing awareness of causes, risk factors, and clinical management of pediatric hearing loss compared to those reflecting perceptions. The mean misconception rate across awareness statements was of 30.4%, with the highest rate observed for the statement that neonatal meningitis or seizures do not cause hearing loss (44%). In contrast, perception-related statements demonstrated a lower mean misconception rate, of 13.4%, with the highest misconception being that parental suspicion of hearing loss should not influence clinical opinion (26%), and the lowest, the belief that hearing loss is not a serious problem in children (5%). These findings indicate that while participants generally acknowledged the importance of pediatric hearing loss, significant gaps remain in the awareness of established medical risk factors and early management practices.

**Table 3:** Misconception percentages for awareness and perception statements about pediatric hearing loss (N = 411)

Statement	Misconception percentage per statement ( *agree* + *strongly agree* responses)
**Awareness statements**	
Craniofacial anomalies (e.g., Down's syndrome) do not cause hearing loss	27%
Intrauterine infections (e.g., cytomegalovirus and rubella) do not cause hearing loss	32%
Low birth weight (< 2.5 kg) does not cause hearing loss	32%
Neonatal jaundice does not cause hearing loss	26%
Neonatal meningitis/seizures does not cause hearing loss	44%
Birth asphyxia does not cause hearing loss	38%
Aminoglycosides such as gentamycin will not harm a baby's hearing	20%
A mother's exposure to loud noise in pregnancy will not affect a baby's hearing	33%
Babies with hearing loss cannot be accurately detected at birth	32%
Hearing aids are unsuitable for babies	20%
**Average misconception rate regarding awareness**	30.4%
**Perception statements**	
Hearing loss is not a serious problem in children	5%
Babies with hearing loss cannot be helped until they are older	14%
Parental suspicion of hearing loss should not influence a clinical opinion	26%
Babies can wait until they are older before testing for hearing loss	10%
Hearing loss is not that important because it does not kill	12%
**Average misconception rate regarding perception**	13.4%

Table 4 presents the Chi-Squared test results on the effect of demographic variables on the participants' awareness and perception. Statistically significant associations were observed between participants' demographic characteristics and their responses to several questionnaire statements ( *p* < 0.05). Age was significantly associated with responses to statements regarding the seriousness of childhood hearing loss, neonatal meningitis or seizures, birth asphyxia, detection at birth, and parental suspicion of hearing loss. Specifically, participants older than 25 years were more likely to disagree that hearing loss is not serious ( *p* = 0.007), while those aged from 18 to 25 years showed stronger awareness of the risks associated with neonatal meningitis ( *p* < 0.001), birth asphyxia ( *p* = 0.009), and intrauterine infections ( *p* = 0.005).

**Table 4:** Effect of demographic variables regarding the combination of *disagree* and *strongly disagree* responses to questions using the Chi-Squared test

Questionnaire statement	Demographic variable	*p* -value
1. Hearing loss is not a serious problem in children	Age (> 25 years); Year of study (year 5); History of hearing loss (yes)	0.007; < 0.001; 0.017
2. Craniofacial anomalies (e.g., Down's syndrome) do not cause hearing loss	Gender (Female); Major (Medical Sciences); Year of study (year 5); History of hearing loss (yes)	< 0.001; < 0.001; < 0.001; 0.04
3. Intrauterine infections (e.g., cytomegalovirus and rubella) do not cause hearing loss	Age (18–25 years); Major (Medical Sciences); Year of study (year 5); History of hearing loss (yes)	0.005; < 0.001; < 0.001; 0.008
4. Low birth weight (< 2.5 kg) does not cause hearing loss	Year of study (year 5)	< 0.001
5. Neonatal jaundice does not cause hearing loss	Major (Medical Sciences); Year of study (year 5)	< 0.001 < 0.001
6. Neonatal meningitis/seizures do not cause hearing loss	Age (18–25 years); Major (Medical Sciences); History of hearing loss (yes)	< 0.001; < 0.001; 0.005
7. Birth asphyxia does not cause hearing loss	Age (18–25 years); Major (Medical Sciences); Year of study (year 3); History of hearing loss (yes)	0.009; 0.007; < 0.001; < 0.001;
8. Aminoglycosides such as gentamycin will not harm a baby's hearing	Gender (Male); Major (Pharmacy); Year of study (year 5)	< 0.001; < 0.001; < 0.001
9. A mother's exposure to loud noise in pregnancy will not affect a baby's hearing	Major (Pharmacy); Year of study (year 5); History of hearing loss (yes)	0.006; < 0.001; < 0.001
10. Babies with hearing loss cannot be accurately detected at birth	Age (18–25 years); Major (Medical Sciences); Year of study (year 4)	< 0.001; 0.004; < 0.001
11. Babies with hearing loss cannot be helped until they are older	Gender (Female); Year of study (year 4)	0.04; < 0.001
12. Hearing aids are unsuitable for babies	Major (Medical Sciences); Year of study (year 4); History of hearing loss (yes)	0.008; < 0.001; 0.013
13. Parental suspicion of hearing loss should not influence a clinical opinion	Age (> 25 years); Year of study (year 5)	< 0.001 < 0.001
14. Babies can wait until they are older before testing for hearing loss	Year of study (year 4)	< 0.001
15. Hearing loss is not that important because it does not kill	Year of study (year 4)	< 0.001

**Note:** Only statistically significant results ( *p* < 0.05) are shown.

Gender was a significant factor for responses to statements about craniofacial anomalies ( *p* < 0.001), with female students showing higher awareness, and for responses about the timing of intervention, in which female students more strongly disagreed that babies cannot be helped until they are older ( *p* = 0.04). Male students were more likely to disagree that aminoglycosides such as gentamycin are harmless to hearing ( *p* < 0.001).

The university major was strongly associated with awareness across multiple risk factors. Medical sciences students showed significantly greater awareness regarding craniofacial anomalies ( *p* < 0.001), intrauterine infections ( *p* < 0.001), neonatal jaundice ( *p* < 0.001), and neonatal meningitis ( *p* < 0.001), among others. Conversely, pharmacy students showed more awareness of the ototoxic effects of gentamycin ( *p* < 0.001) and the impact of maternal exposure to noise ( *p* = 0.006).

The year of study had a strong influence on responses to nearly all awareness-related statements, with year-5 students consistently showing higher awareness, for example, about the seriousness of hearing loss ( *p* < 0.001), craniofacial anomalies ( *p* < 0.001), and intrauterine infections ( *p* < 0.001). Awareness also improved significantly by year 4 for statements related to early detection and intervention ( *p* < 0.001).

A personal or family history of hearing loss was significantly associated with higher awareness in several areas, including the seriousness of pediatric hearing loss ( *p* = 0.017), craniofacial anomalies ( *p* = 0.04), and birth asphyxia ( *p* < 0.001).

## Discussion

The present study revealed that, while health sciences students broadly recognize pediatric hearing loss as a serious condition, there are significant gaps in their clinical knowledge. Most students expressed support for early hearing testing in children, but their awareness of the underlying causes of hearing loss was limited. Many respondents demonstrated uncertainty or incorrect beliefs about well-established risk factors. Thus, the study findings suggested that students were not sufficiently prepared to recognize or act on the clinical risks contributing to pediatric hearing loss in their future practice.

Most students supported the concept of early hearing testing in children, reflecting positive attitudes toward proactive screening and early identification. However, despite these generally-favorable views, notable gaps emerged in their knowledge of specific etiologies of hearing loss. Many respondents were unsure or misinformed about the potential impact of neonatal jaundice (severe hyperbilirubinemia), ototoxic neonatal medications such as gentamycin, or even maternal exposure to noise during pregnancy. This uncertainty suggested that while students appreciate the importance of pediatric hearing loss in principle, they lacked clarity regarding the diverse risk factors for hearing loss. The gap between positive perceptions and inadequate factual knowledge may hinder the timely identification and management of hearing loss in children. 18

Our findings aligned with and extended the observations from studies across the Middle East and other international contexts regarding healthcare students' awareness of pediatric hearing loss. In the Gulf Cooperation Council countries, 16 university healthcare students demonstrated limited knowledge of pediatric hearing loss causes, such as birth complications, despite recognizing the importance of early detection. Limited exposure to pediatric audiology during undergraduate training was noted as a key barrier to improving knowledge and referral practices in the Middle East. 19 20 Similar patterns have been observed globally. In Nigeria, 14 for example, final-year medical students and practicing physicians showed limited knowledge of key risk factors for infant hearing loss, with only 11 to 43% recognizing factors such as low birth weight or maternal exposure to noise. Similarly, in Samoa, 13 only about half of nursing students were aware of the importance of early detection, and even fewer knew that infants could be fitted with amplification devices. In the United States, Squires et al. 21 (2019) found that while healthcare students had greater awareness of general hearing loss, only half of them correctly identified the recommended age for newborn hearing screening and early intervention. These international findings reflect similar knowledge gaps in our Jordanian sample, pointing to a pattern of positive attitudes but limited awareness of its causes and management.

The current analysis also showed that demographic and training-related factors significantly influenced awareness levels. Older students and those in later academic years demonstrated higher awareness, which is consistent with studies from Nigeria 14 and India 15 showing greater knowledge about risk factors among more experienced medical trainees. Gender differences, with female students scoring higher in certain domains, were also observed in the present study. Similar findings have been reported in previous research, 22 and they may reflect contextual factors, such as gendered learning experiences or socialization around caregiving roles. Finally, students with a family history of hearing loss were significantly more knowledgeable, a finding echoed in general population surveys 18 showing that personal exposure may enhance awareness. These patterns underscored that academic exposure and personal experience contribute meaningfully to how students perceive and understand pediatric hearing loss.

The findings of the current study carry significant implications for health professional education and pediatric hearing loss initiatives in Jordan and similar contexts. First and foremost, the apparent knowledge gaps identified suggest a need to strengthen pediatric hearing health content in the curricula of health sciences programs. Key topics such as universal newborn hearing screening, risk factors for congenital and early-onset hearing loss, and early intervention protocols should be introduced earlier and reinforced throughout training. Many studies supported that embedding these topics into core curricula enhances awareness and readiness to engage in early detection and referral practices. 13 14 19 When students are confident in identifying risk factors and advocating for referrals, the likelihood of timely diagnosis and intervention increases, ultimately improving developmental outcomes for children. 23

The present study is not without limitations. Data were collected from a single institution, which may limit generalizability and introduce sampling bias. Participation was voluntary, creating the possibility of response bias toward students with greater interest or background in hearing health; at the same time, the high response rate strengthens the reliability of the findings. The use of self-reported measures may also be influenced by social desirability bias, in which students provide answers they believe to be correct, and it captures perceived rather than objectively-tested knowledge. Nevertheless, self-reported surveys remain a widely-used and efficient method to capture large-scale data on attitudes and knowledge. Despite these limitations, the study offers valuable insight into university students' understanding of pediatric hearing loss in Jordan, it contributes novel evidence from a Middle Eastern context, and it highlights important areas for future research and curriculum development.

## Conclusions

The current study revealed that health sciences students recognize the seriousness of pediatric hearing loss; however, their knowledge of its causes and management remains limited. Awareness was particularly low for validated risk factors, and knowledge varied by age, academic level, field of study, and family history. These findings highlight the need to strengthen pediatric hearing health education in health sciences programs to ensure students are better prepared for early identification and referral of childhood hearing loss.
